# Sphingomonas paucimobilis bacteremia and tricuspid valve endocarditis in a patient with intravenous drug use

**DOI:** 10.1016/j.idcr.2022.e01399

**Published:** 2022-01-10

**Authors:** Wesley Tang, Sulagna Das, Satish Sarvepalli

**Affiliations:** a Department of Medicine, Kettering Medical Center, Kettering, OH, USA; b Division of Infectious Disease, South Dayton Acute Care Consultants, Dayton, OH, USA

**Keywords:** Sphingomonas paucimobilis, Bacteremia, Endocarditis, IV drug use

## Abstract

*Sphingomonas paucimobilis* is an aerobic, yellow-pigmented, glucose non-fermenting, gram negative bacillus that is a rare cause of human infection found mostly in the immunocompromised and also in intravenous (IV) drug users. We report a case of a 31-year-old female with current IV drug use, who presented with chest pain and was diagnosed with tricuspid valve endocarditis with *S. paucimobilis* bacteremia and pulmonary infarction of the right middle lobe. The patient initially presented with sharp right sided chest pain. She was treated with meropenem and levofloxacin based on the susceptibility profile. Our purpose is to highlight the treatment options and raise awareness of this uncommon organism. Even though Sphingomonas is considered to be of low-pathogenicity, it can be fatal if not treated properly and not diagnosed early.

## Introduction

*Sphingomonas paucimobilis* is a Gram-negative, strictly aerobic, non-spore-forming bacillus that is found widely distributed in the soil and water [1]. It has also been isolated in sterile water in hospitals, indwelling medical devices, ventilators, catheters, and central venous lines [2], [3]. The genus *Sphingomonas* contains at least 12 species, of which only *S. paucimobilis* is considered a potential human pathogen [4]. Sphingomonas is considered to be of low-pathogenicity, and is regarded as an opportunistic microorganism. Reports of *S. paucimobilis* infection have included pneumonia, catheter-associated infections, osteomyelitis, septic arthritis, splenic abscesses, urinary tract infections, and biliary tract infections [5]. Cases of *Sphingomonas* bacteremia are few, with cases of infective endocarditis even fewer. To date, a literature review demonstrated only 1 case of *S. paucimobilis* endocarditis in a native valve reported in the United States, and 3 other reported cases in Mexico, China, and Taiwan [6]. We detail only the second reported case of *Sphingomonas paucimobilis* tricuspid valve endocarditis, with associated bacteremia, and pulmonary infarction to the right middle lobe, in a patient with active intravenous (IV) drug use.

## Case description

**Fig. 1:**
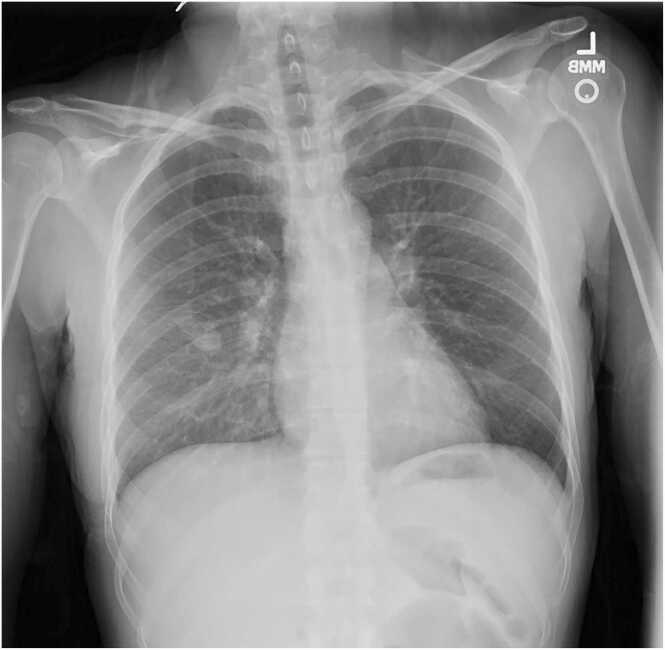
Initial chest xray image with mild right perihilar infiltrate and right middle lobe lung nodule.

**Fig. 2:**
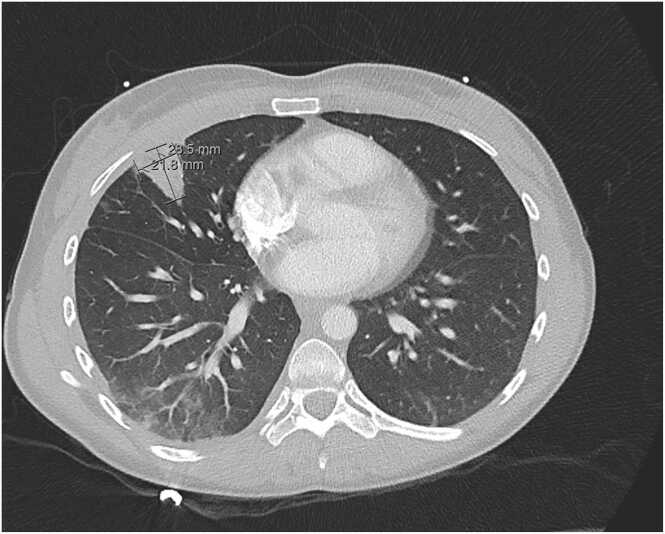
Computed tomography image with a wedge-shaped right middle lobe lung nodule.

A 31-year-old female with history of IV drug use and chronic hepatitis C presented to our hospital with a 24-hour history of sharp right-sided chest pain which worsened with movement and deep inspiration. She denied fever, chills, chest trauma, falls, or injury. She reported that she uses heroin every 2–3 days mostly by injection and occasionally by snorting. Initial chest x-ray was concerning for right perihilar infiltrate and right middle lobe nodule, both suggestive of pneumonia (Fig. 1). Follow up computed tomography with pulmonary embolism protocol showed right sided pulmonary infarction with mediastinal and bilateral hilar lymph nodes (Fig. 2). Complete blood counts and basic metabolic panels were unremarkable and vitals were within normal limits. As there was concern for septic emboli, cardiology was consulted for transesophageal echocardiography (TEE), which revealed a hypermobile, small mass on the leaflet tip of the tricuspid valve suggestive of infective endocarditis. Empiric vancomycin was started pending blood cultures and Infectious disease consult was requested. On day 3 of hospital admission, the patient developed septic shock with fever, severe hypotension and tachycardia. Blood cultures grew gram-negative rods and the patient was started on meropenem 500 mg IV every 6 h, and vancomycin was discontinued. The following day, blood cultures resulted in *Sphingomonas paucimobilis* with the isolate susceptible to meropenem, levofloxacin, aminoglycosides, and tetracyclines but resistant to piperacillin/tazobactam. Oral levofloxacin 750 mg daily was added to meropenem for dual antimicrobial coverage for gram-negative bacteremia per ID recommendations. The patient’s clinical condition improved significantly over the next several days. Once repeat blood cultures were negative, a peripherally inserted central catheter (PICC) line was placed and the patient was discharged on meropenem and levofloxacin for a total planned course of 6 weeks of antimicrobial therapy. The patient was recommended to follow up with cardiology in 6 weeks for repeat TEE for re-evaluation of the tricuspid valve following completion of antibiotic therapy. Unfortunately, the patient has been lost to follow up since hospital discharge.

## Discussion

*Sphingomonas paucimobilis* can cause a variety of infections and is most often associated with nosocomial infections, especially in patients with indwelling intravascular devices [5]. *S. paucimobilis* is an organism of low virulence and is uncommonly isolated in the immunocompetent. The authors postulate that our patient with community-acquired *S. paucimobilis* self-inoculated as a consequence of ongoing intravenous drug use, leading to bacteremia and infective tricuspid endocarditis.

To date there have been no established guidelines in the treatment of *S. paucimobilis*, in particular, invasive disease like endocarditis. Isolated case reports have found success with tetracycline, trimethoprim/sulfamethoxazole, carbapenems, and aminoglycosides [7]. In one literature review of published case reports, other antibiotics that have shown to have activity against *S. paucimobilis,* include ampicillin/sulbactam, fluoroquinolones, and piperacillin/tazobactam [5]. Our particular isolate was found to be resistant to cefazolin and piperacillin/tazobactam, sensitive to aminoglycosides and fluoroquinolones, and no carbapenems were tested. Based on the above literature review and in conjunction with our isolate susceptibilities, we opted to treat our patient with dual gram-negative coverage with both meropenem and levofloxacin for a total of 6 weeks. Even with inappropriate antimicrobial therapy, the overall outcome for patients is favorable, with a survival rate of 95.2% [8].

In conclusion, this rare case of community-acquired *S. paucimobilis* bacteremia with associated endocarditis can occur in patients who use intravenous drugs, and not just immunocompromised hosts. This case represents only the second case of bacteremia and endocarditis reported in the United States. The patient successfully completed the recommended course of meropenem and levofloxacin. Unfortunately the patient was lost to follow up and did not complete a TEE scheduled at 6 weeks from initiation of antimicrobial therapy. This organism should be considered as a ubiquitous pathogen, and not just a nosocomial contaminant. It is likely that this organism is under-reported and under-recognized.

## Funding support

None.

## Consent

Written informed consent was obtained from the patient for publication of this case report and accompanying images. A copy of the written consent is available for review by the Editor-in-Chief of this journal on request.

## CRediT authorship contribution statement

Wesley Tang, Sulagna Das, Satish Sarvepalli designed and conducted the research. Satish Sarvepalli provided the data. Wesley Tang had primary responsibility for the final content. All authors read and approved the final manuscript.

## Conflicts of interest statement

The authors declare that they do not have a conflict of interest.
